# P05.25. Exploring the safety culture in spinal manipulative therapy: interviews with key informants and frontline practitioners

**DOI:** 10.1186/1472-6882-12-S1-P385

**Published:** 2012-06-12

**Authors:** H Boon, S Mior, L Rozmovits

**Affiliations:** 1 Leslie Dan Faculty of Pharmacy, University of Toronto, Toronto, Ontario, Canada; 2 Canadian Memorial Chiropractic College, Toronto, Canada

## Purpose

Recent surveys estimate that 50% of Canadians have received spinal manipulation therapy (SMT), most commonly for back and neck pain. Despite its popularity, no formal safety reporting and learning mechanisms exist to allow Canadian regulated health professions who provide SMT to monitor and reduce related harms. This study explores what stakeholders in Alberta perceive to be the barriers to, and opportunities for, the development of a safety culture in SMT.

## Methods

This comparative case study involved: (1) key informants from regulating bodies, professional associations and training facilities of the four professional groups that include SMT in their scope of practice in Canada (chiropractors, physicians, physiotherapists and osteopaths; n=12-15); and (2) practitioners from each discipline who undertake SMT in their practices (n=30-40) and practice in Alberta, Canada. Semi-structured telephone interviews were conducted with each participant. The interviews were transcribed verbatim and subjected to content analysis. Interviews are expected to be completed by December 2011.

## Results

While patient safety was a widely expressed priority, conventional safety culture concepts (such as “no blame, no shame”) were largely unfamiliar to participants. There were also fears about how “safety” information might be used against practitioners who offer SMT. Structural factors such as combined professional associations and regulatory colleges and the influence of professional protective associations on the safety culture emerged, but there was limited understanding of how they may influence patient safety. Ongoing interviews will probe these themes further and explore differences between professional groups as well as between key informants and frontline practitioners.

## Conclusion

The findings will be used to inform the development of a spinal manipulation safety culture intervention that will be implemented in the second phase of this project.

